# In-Hospital Outcomes According to Treatment Strategy in NSTEMI Patients Undergoing Coronary Angiography: No Index Revascularization, PCI, or CABG?

**DOI:** 10.3390/jcm15207872

**Published:** 2026-10-13

**Authors:** Kemal Eşref Erdoğan, Muhammet Fethi Sağlam, Serdal Baştuğ, Burak Kardeşler, Emrah Uğuz, Etga Köprücü, Murat Yücel, Mehmet Murat Yiğitbaşı, Kamuran Kalkan, Hüseyin Bayram, Murat Akçay

**Affiliations:** 1 Department of Cardiovascular Surgery, Faculty of Medicine, Ankara Yildirim Beyazit University, Ankara 06800, Türkiye; dr.m.fethisaglam@gmail.com (M.F.S.); emrahuguz@gmail.com (E.U.); 2 Department of Cardiovascular Surgery, Ankara Bilkent City Hospital, Ankara 06800, Türkiye; dr_yucelmurat@hotmail.com (M.Y.); drhuseyinbayram@gmail.com (H.B.); 3 Department of Cardiology, Faculty of Medicine, Ankara Yildirim Beyazit University, Ankara 06800, Türkiye; serdalbastug@yahoo.com (S.B.); mmuratyigitbasi@gmail.com (M.M.Y.); kamurankalkandr@gmail.com (K.K.); drmuratakcay@yahoo.com (M.A.); 4 Department of Cardiology, Ankara Bilkent City Hospital, Ankara 06800, Türkiye; burak_kardesler@hotmail.com (B.K.); etgakprc07@gmail.com (E.K.)

**Keywords:** NSTEMI, no index revascularization, percutaneous coronary intervention, coronary artery bypass grafting, in-hospital outcomes, MACE, multiple imputation

## Abstract

**Background/Objectives**: Real-world data comparing treatment strategies and in-hospital outcomes across no index revascularization, percutaneous coronary intervention (PCI), and coronary artery bypass grafting (CABG) in NSTEMI patients remain limited. **Methods**: In this single-center retrospective cohort study, 1906 NSTEMI patients undergoing coronary angiography were categorized into no index revascularization (*n* = 946), PCI (*n* = 709), or CABG (*n* = 251) groups. The primary endpoint was in-hospital major adverse cardiovascular events (MACE), defined as all-cause mortality, cardiac arrest requiring cardiopulmonary resuscitation, sustained ventricular tachycardia/ventricular fibrillation, dialysis-requiring acute kidney injury, or stroke. Missing data were handled using multiple imputation with chained equations (MICE), and propensity score-based inverse probability of treatment weighting (IPTW) was used as a sensitivity analysis. **Results**: In-hospital MACE was higher in the no index revascularization group than in the PCI and CABG groups (9.8% vs. 3.5% vs. 4.8%; *p* < 0.001). Patients managed with no index revascularization were older and had a greater comorbidity burden, lower left ventricular ejection fraction, and more frequent Killip class ≥ 2. In the primary multivariable model, chronic kidney disease, Killip class ≥ 2, lower LVEF, CRP, and male sex were associated with in-hospital MACE, whereas neither PCI nor CABG strategy was independently associated with MACE. IPTW sensitivity analyses yielded similar directions of association; however, the PCI estimate crossed the conventional significance threshold after 1st–99th percentile weight truncation. **Conclusions**: Patients managed with no index revascularization had a higher baseline risk profile and higher unadjusted in-hospital MACE and mortality. The observed between-group differences should be interpreted as associations rather than causal effects of treatment strategy because of substantial confounding by indication.

## 1. Introduction

Acute coronary syndromes (ACS) remain a leading cause of cardiovascular mortality and morbidity worldwide [1]. Non-ST-elevation myocardial infarction (NSTEMI) represents the most common form of ACS, accounting for approximately half of all ACS cases. Unlike ST-elevation myocardial infarction (STEMI), NSTEMI encompasses a heterogeneous patient population, making both risk stratification and optimal treatment strategy selection particularly challenging [2].

Current guidelines from the European Society of Cardiology (ESC) and the American College of Cardiology/American Heart Association (ACC/AHA) recommend an invasive approach in appropriately selected patients with NSTEMI, with subsequent revascularization decisions individualized according to coronary anatomy, clinical characteristics, comorbidity burden, and procedural risk [3,4,5]. Importantly, coronary angiography does not necessarily lead to revascularization, and no index revascularization may represent an appropriate management pathway after angiographic assessment in selected patients. In real-world practice, a substantial proportion of patients undergoing coronary angiography for NSTEMI are managed with no index revascularization, whereas others undergo PCI or are referred for surgical revascularization [6].

While PCI is the most frequently performed revascularization strategy in NSTEMI, CABG may be the preferred option in the presence of left main coronary artery (LMCA) disease, multivessel disease, or high SYNTAX scores [7]. Conversely, patients with significant comorbidities, unfavorable coronary anatomy, or high procedural risk may not undergo either percutaneous or surgical revascularization and may instead be managed with no index revascularization after coronary angiography [8,9].

Randomized controlled trials have provided important evidence on NSTEMI management; however, strict inclusion and exclusion criteria limit their generalizability to everyday clinical practice [10,11]. Real-world cohort studies capturing broader, more heterogeneous patient populations offer a unique opportunity to examine clinical outcomes associated with different treatment pathways under routine clinical conditions. Comparative analyses of in-hospital outcomes across no index revascularization, PCI, and CABG groups in real-world NSTEMI cohorts are scarce [9].

In this study, we aimed to characterize baseline differences and compare in-hospital clinical outcomes across three treatment strategies—no index revascularization, PCI, and CABG—in NSTEMI patients undergoing coronary angiography in a real-world setting. We also sought to identify independent predictors of in-hospital MACE using multivariable logistic regression with multiple imputation to account for missing data.

## 2. Materials and Methods

### 2.1. Study Design and Patient Population

This was a single-center, retrospective cohort study. Consecutive patients admitted to the cardiology intensive care unit between January 2020 and December 2025 with a diagnosis of NSTEMI and who underwent coronary angiography (CAG) at the index hospital were retrospectively evaluated. The study was conducted in accordance with the Declaration of Helsinki, and Ethics Committee approval was obtained on 11 March 2026 (protocol code: TABED 1-2300-2026) before retrospective data extraction and analysis were initiated.

**Figure 1:**
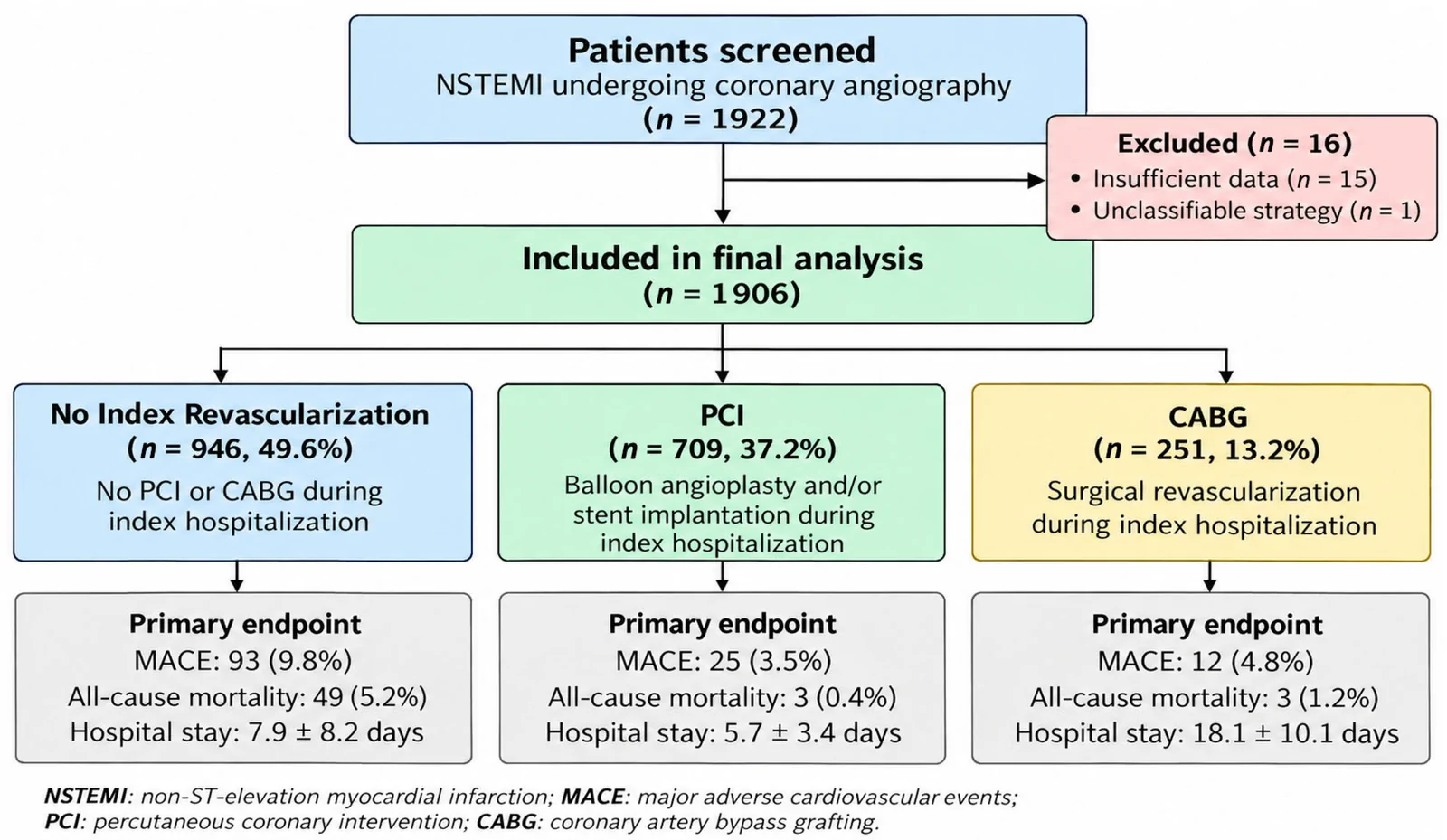
Patient flow diagram. Hospital-stay values are based on available observations (no index revascularization, *n* = 240; PCI, *n* = 176; CABG, *n* = 57).

Patients meeting all the following criteria were included: (1) NSTEMI diagnostic criteria (typical chest pain or equivalent symptoms (e.g., dyspnea, diaphoresis, nausea/vomiting, or unexplained weakness) with cardiac troponin elevation and new ST depression, T-wave changes, or a normal electrocardiogram); and (2) coronary angiography performed during the index hospitalization. NSTEMI was diagnosed in accordance with the Fourth Universal Definition of Myocardial Infarction, requiring a rise and/or fall in cardiac troponin with at least one value above the assay-specific 99th-percentile upper reference limit, together with clinical evidence of acute myocardial ischemia. Patients with type 2 myocardial infarction and non-ischemic myocardial injury were excluded based on the overall clinical assessment [12]. MINOCA was defined as myocardial infarction fulfilling the diagnostic criteria for acute MI in the absence of obstructive coronary artery disease, with no coronary stenosis ≥50% in any major epicardial coronary artery. MINOCA patients were retained in the primary cohort because treatment groups were defined according to the final in-hospital management pathway after coronary angiography. Accordingly, patients with MINOCA who underwent neither PCI nor CABG were classified in the no index revascularization group. Given the potential clinical heterogeneity of this group, a sensitivity analysis excluding patients with MINOCA was performed. Of 1922 screened patients, 15 with insufficient data and 1 with an unclassifiable treatment strategy were excluded, leaving 1906 patients for the final analysis (Figure 1).

### 2.2. Treatment Groups

Patients were categorized into three groups according to the final in-hospital management pathway after coronary angiography: (1) no index revascularization group—patients who underwent neither PCI nor CABG during the index hospitalization (*n* = 946); (2) PCI group—patients who underwent balloon angioplasty and/or coronary stent implantation during the index hospitalization (*n* = 709); and (3) CABG group—patients who underwent surgical revascularization during the index hospitalization (*n* = 251). The PCI group included both single session multivessel PCI and staged PCI approaches. Revascularization decisions were made by the Heart Team or the responsible cardiologist based on coronary anatomy, clinical presentation, and patient preference. Treatment groups were defined retrospectively according to the final management pathway during the index hospitalization rather than according to an intention-to-treat strategy at the time of angiography. Therefore, the time of treatment allocation was not used as a common time zero for outcome assessment.

### 2.3. Data Collection and Variables

Patient data were retrospectively collected from electronic medical records and catheterization laboratory reports. Demographic characteristics, cardiovascular risk factors, comorbidities, admission heart rate, Killip class, echocardiographic parameters (left ventricular ejection fraction [LVEF]), and laboratory values (serum creatinine, estimated glomerular filtration rate [eGFR], C-reactive protein [CRP], hemoglobin, and peak cardiac troponin) were recorded. Angiographic data included the number of diseased vessels, LMCA involvement, and time to angiography. For CABG patients, additional variables including time to surgery, number of grafts, graft types, and target vessels were recorded. Because angiographic variables were retrospectively extracted from catheterization reports and electronic records, structured documentation of LMCA and multivessel disease was incomplete in a proportion of patients. When reliable vessel-level quantification could not be extracted, these variables were coded as missing rather than inferred from other clinical data. MINOCA status was based on the dedicated clinical/angiographic classification recorded in the source data and was not reconstructed solely from the LMCA or multivessel-disease variables. Missing LMCA and multivessel-disease values were handled according to the prespecified missing-data procedures used in the respective multivariable analyses. eGFR values were extracted directly from the electronic laboratory records when available and were not retrospectively calculated from serum creatinine. Peak troponin represented high-sensitivity cardiac troponin I (hs-cTnI) and was recorded in ng/mL.

### 2.4. Outcome Definitions

The primary endpoint was in-hospital MACE, defined as a composite of: (1) all-cause mortality; (2) cardiac arrest requiring cardiopulmonary resuscitation (CPR); (3) sustained ventricular tachycardia or ventricular fibrillation (VT/VF); (4) dialysis-requiring acute kidney injury (AKI); and (5) stroke during the index hospitalization. For the purposes of this retrospective study, outcome events were identified from documented clinical diagnoses and treatment records during the index hospitalization. Dialysis-requiring AKI was defined operationally as acute renal deterioration requiring initiation of renal replacement therapy during the index hospitalization. Sustained VT/VF was defined as documented ventricular tachycardia or ventricular fibrillation requiring acute clinical intervention during the index hospitalization. Stroke was defined as a new focal neurological deficit documented as an acute stroke in the clinical record during the index hospitalization. In-hospital atrial fibrillation was defined as atrial fibrillation documented as a clinical diagnosis in the medical record during the index hospitalization.

Secondary endpoints included each individual component of MACE, in-hospital bleeding events (intracranial hemorrhage, gastrointestinal bleeding, genitourinary bleeding, groin hematoma, or transfusion-requiring bleeding), in-hospital atrial fibrillation, and total hospital stay. Bleeding was therefore analyzed as a study-defined in-hospital bleeding endpoint rather than according to a retrospectively assigned BARC classification. Given the heterogeneous nature of its components, this study-specific composite should not be interpreted as a conventional cardiovascular MACE endpoint, and its individual components were also evaluated separately. All events were verified by reviewing clinical records and daily patient follow-up notes.

### 2.5. Statistical Analysis

Continuous variables are expressed as mean ± standard deviation (normally distributed) or median [interquartile range] (skewed distribution). Categorical variables are reported as number and percentage (%). Between-group comparisons of continuous variables were performed using one-way analysis of variance or the Kruskal–Wallis test, as appropriate. Categorical variables were compared using the chi-square test or Fisher’s exact test. All tests were two-sided, and *p* < 0.05 was considered statistically significant.

To identify independent predictors of in-hospital MACE, multivariable logistic regression was performed. Missing covariate data were handled using multiple imputation with chained equations implemented with the IterativeImputer procedure in scikit-learn. Twenty imputed datasets were generated using Bayesian ridge regression with posterior sampling. Ten chained-equation iterations were performed for each imputation, and fixed random seeds were used to ensure reproducibility. Continuous covariates were imputed on their observed scale, whereas imputed binary covariates were constrained to binary values using a threshold of 0.5. The MACE outcome and treatment-group indicators were included as predictors in the imputation procedure but were not imputed. Multivariable logistic regression was fitted separately in each of the 20 imputed datasets, and regression coefficients and standard errors were combined using Rubin’s rules. Stability of the imputation procedure was assessed by repeating the analysis with 20 chained-equation iterations; the pooled regression estimates were materially unchanged. Covariates were selected a priori based on established clinical relevance to short-term NSTEMI outcomes and potential association with treatment selection, while considering the number of observed MACE events. With 130 MACE events and 12 covariates included in the final model, the event-to-variable ratio was approximately 10.8, which is at the lower end of conventionally accepted values for multivariable logistic regression. Multicollinearity was assessed using variance inflation factors (VIFs). Because peak troponin was missing in 866 of 1906 patients (45.4%) and represented the maximum concentration recorded during hospitalization, it was excluded from the primary multivariable model and evaluated separately in a sensitivity analysis using the same multiple-imputation procedure. A complete-case analysis including peak troponin was also performed. For model-performance assessment, predicted probabilities were calculated in each of the 20 imputed datasets using the pooled regression coefficients and then averaged across imputations for each patient. The c-statistic, Brier score, and Hosmer–Lemeshow goodness-of-fit statistic were calculated from these averaged patient-level predicted probabilities.

To further address confounding by indication, propensity score-based inverse probability of treatment weighting (IPTW) was performed as a sensitivity analysis. Pairwise propensity-score models were constructed separately for PCI versus no index revascularization and CABG versus no index revascularization. Peak troponin was not included in the propensity score models because the recorded value represented the peak value during hospitalization and could therefore occur after treatment allocation. The IPTW analyses were restricted to pretreatment covariates. The propensity score models included age, sex, hypertension, diabetes mellitus, chronic obstructive pulmonary disease, chronic kidney disease, previous myocardial infarction, current smoking, Killip class ≥2, heart rate, left ventricular ejection fraction, serum creatinine, estimated glomerular filtration rate, C-reactive protein, hemoglobin, left main coronary artery disease, and multivessel disease. Missing values in propensity-score covariates were imputed using the median of the available observations. Stabilized average-treatment-effect weights were used without weight truncation. Patients outside the region of common support were retained in the weighted analyses; no observations were excluded based on common support. Propensity-score overlap, weight distributions, effective sample size (ESS), and covariate balance before and after weighting were examined. Covariate balance was assessed using standardized mean differences (SMDs), with an absolute SMD <0.10 considered indicative of adequate balance. Weighted logistic regression was used to evaluate treatment-strategy associations with in-hospital MACE. Given the more limited overlap and residual covariate imbalance in the CABG comparison, the CABG IPTW analysis was considered exploratory. Accordingly, the regression and IPTW analyses were interpreted as adjusted associational analyses rather than estimates of causal treatment effects. Analyses were performed using Python 3.13.5 with scikit-learn 1.8.0 (IterativeImputer), statsmodels 0.14.6, and SciPy 1.17.0.

As an additional sensitivity analysis, stabilized IPTW weights were truncated at the 1st and 99th percentiles, and the weighted treatment-strategy models were re-estimated.

## 3. Results

### 3.1. Patient Characteristics and Treatment Groups

A total of 1906 NSTEMI patients undergoing coronary angiography were included. Patients were categorized into three treatment groups: no index revascularization (*n* = 946, 49.6%), PCI (*n* = 709, 37.2%), and CABG (*n* = 251, 13.2%). Baseline demographic, clinical, and angiographic characteristics are presented in Table 1.

Patients in the no index revascularization group were significantly older than those in the PCI and CABG groups (67.0 ± 13.9 vs. 59.7 ± 11.0 vs. 60.7 ± 10.0 years; *p* < 0.001). The proportion of male patients was significantly lower in the no index revascularization group than in the PCI and CABG groups (56.9% vs. 78.6% vs. 74.5%, respectively; *p* < 0.001).

The no index revascularization group had higher prevalences of hypertension (72.1% vs. 53.6% vs. 61.4%; *p* < 0.001), diabetes mellitus (43.4% vs. 34% vs. 38.6%; *p* < 0.001), COPD (10.3% vs. 4.7% vs. 4.2%; *p* < 0.001), CKD (19.9% vs. 5.7% vs. 4.2%; *p* < 0.001), and previous MI (36.8% vs. 26.3% vs. 19.0%; *p* < 0.001). Killip class ≥2 at presentation was most frequent in the no index revascularization group (14.7% vs. 3.1% vs. 3.4%; *p* < 0.001). Smoking was most common in the PCI group (48.5% vs. 28.0% vs. 39.4%; *p* < 0.001).

The no index revascularization group had lower LVEF (47.3 ± 12.1 vs. 52.1 ± 9.4 vs. 52.0 ± 9.6%; *p* < 0.001), higher heart rate (90.8 ± 25.7 vs. 80.8 ± 19.4 vs. 84.4 ± 24.5 bpm; *p* < 0.001), higher serum creatinine (1.37 ± 1.25 vs. 1.02 ± 0.86 vs. 0.98 ± 0.42 mg/dL; *p* < 0.001), lower eGFR (69.0 ± 30.4 vs. 85.7 ± 20.6 vs. 85.5 ± 20.1 mL/min/1.73 m^2^; *p* < 0.001), higher CRP (3.3 ± 4.8 vs. 1.8 ± 3.0 vs. 2.6 ± 4.3 mg/L; *p* < 0.001), and lower hemoglobin (12.8 ± 2.2 vs. 14.1 ± 1.7 vs. 13.6 ± 1.9 g/dL; *p* < 0.001).

Angiographically, LMCA disease (3.5% vs. 1.7% vs. 16.8%; *p* < 0.001) and multivessel disease (33.1% vs. 33.2% vs. 89.9%; *p* < 0.001) were most prevalent in the CABG group. Time-to-angiography data were available for only 373 of 1906 patients (19.6%): 142/946 in the no index revascularization group, 175/709 in the PCI group, and 56/251 in the CABG group. Among these available cases, mean time to angiography was 65.9 ± 72.2, 48.4 ± 46.9, and 29.9 ± 37.5 h, respectively. Given the substantial missingness, this comparison should be interpreted cautiously and is descriptive only.

### 3.2. In-Hospital Outcomes

**Figure 2:**
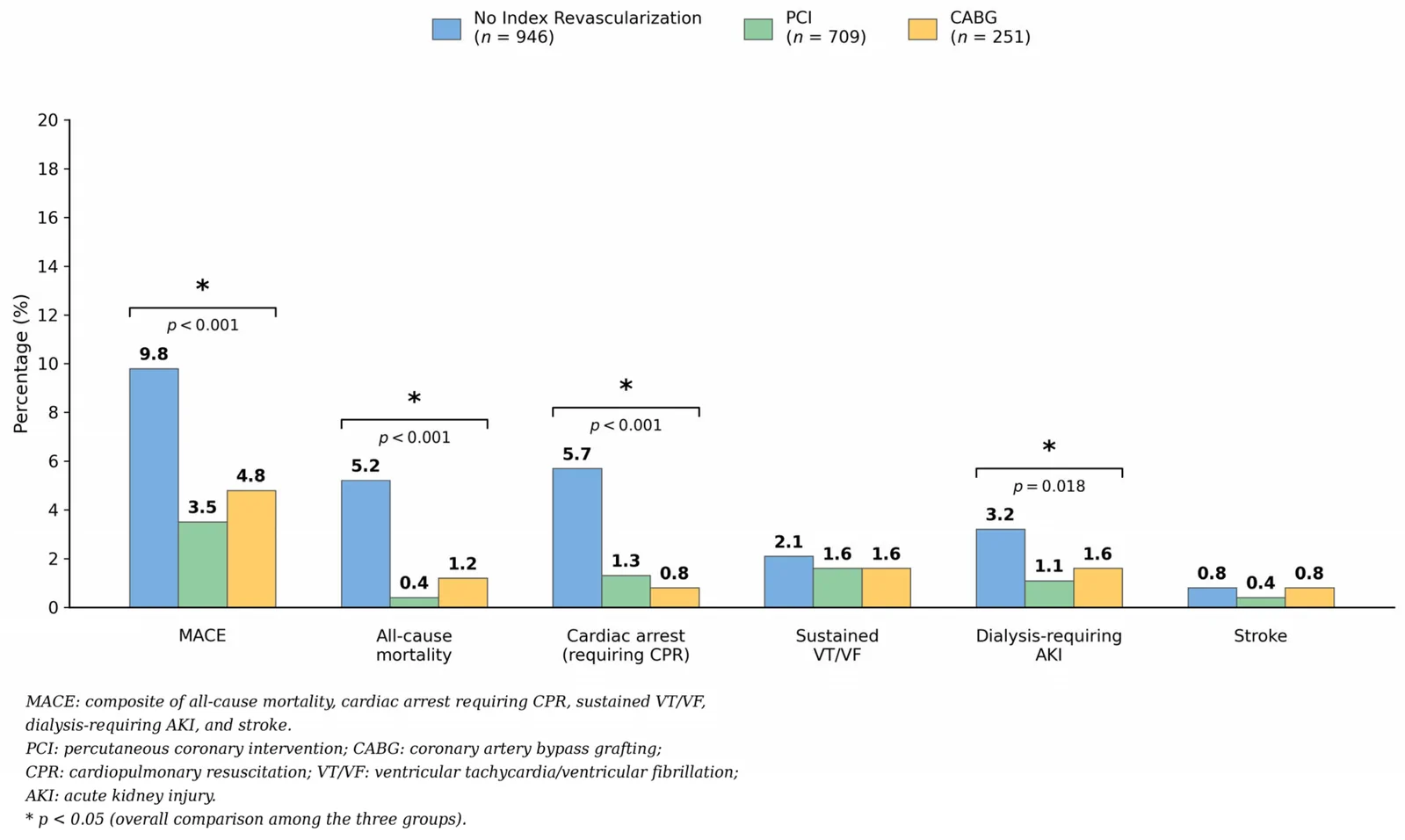
In-hospital outcomes by treatment strategy.

In-hospital outcomes are summarized in Table 2 and Figure 2. The primary composite endpoint of in-hospital MACE occurred significantly more frequently in the no index revascularization group compared to PCI and CABG groups (9.8% vs. 3.5% vs. 4.8%; *p* < 0.001). All-cause mortality was markedly higher in the no index revascularization group (5.2% vs. 0.4% vs. 1.2%; *p* < 0.001), as were cardiac arrest requiring CPR (5.7% vs. 1.3% vs. 0.8%; *p* < 0.001) and dialysis-requiring AKI (3.2% vs. 1.1% vs. 1.6%; *p* = 0.018). Sustained VT/VF (2.1% vs. 1.6% vs. 1.6%; *p* = 0.742) and stroke (0.8% vs. 0.4% vs. 0.8%; *p* = 0.620) did not differ significantly across groups. In an exploratory sensitivity analysis excluding sustained VT/VF and dialysis-requiring AKI from the composite endpoint, event rates remained significantly different among the no index revascularization, PCI, and CABG groups (6.6%, 1.7%, and 2.4%, respectively; *p* < 0.001).

In-hospital bleeding events were uncommon in the no index revascularization and PCI groups (0.5% and 0.1%, respectively; *p* = 0.247). In-hospital atrial fibrillation occurred in 4.3% and 1.0% of patients in the no index revascularization and PCI groups, respectively (*p* < 0.001). CABG-group bleeding and atrial fibrillation data are not presented because these secondary outcomes may have been underestimated in the retrospective dataset. Total hospital stay data were available for 240, 176, and 57 patients in the no index revascularization, PCI, and CABG groups, respectively. These partial-sample data are presented descriptively only; mean total hospital stay was 7.9 ± 8.2, 5.7 ± 3.4, and 18.1 ± 10.1 days, respectively.

### 3.3. Multivariable Logistic Regression Analysis for In-Hospital MACE

In the primary model, CKD (OR 3.70; 95% CI 2.36–5.81; *p* < 0.001), lower LVEF (OR 0.97; 95% CI 0.95–0.99; *p* = 0.003), Killip class ≥ 2 (OR 2.20; 95% CI 1.30–3.71; *p* = 0.003), CRP (OR 1.06; 95% CI 1.02–1.10; *p* = 0.003), and male sex (OR 1.63; 95% CI 1.04–2.58; *p* = 0.035) were independently associated with in-hospital MACE. Neither PCI (OR 0.68; 95% CI 0.41–1.13; *p* = 0.137) nor CABG (OR 0.92; 95% CI 0.42–2.00; *p* = 0.833) was independently associated with MACE compared with no index revascularization. The model showed good discrimination (c-statistic = 0.808) and a Brier score of 0.055. The Hosmer–Lemeshow goodness-of-fit test showed no evidence of poor calibration (χ^2^ = 12.56, df = 8; *p* = 0.128) (Table 3).

### 3.4. Propensity Score-Based Sensitivity Analysis

In the pairwise IPTW sensitivity analysis comparing PCI with no index revascularization, PCI was not significantly associated with in-hospital MACE (OR 0.71, 95% CI 0.48–1.06; *p* = 0.097). Propensity-score overlap was high, with approximately 0.5% of patients outside the common support. The overall weighted effective sample size was 908. Covariate balance improved substantially after weighting; however, minor residual imbalance remained for Killip class ≥ 2 (SMD = 0.115) and CKD (SMD = 0.100). In the CABG versus no index revascularization analysis, CABG was also not significantly associated with in-hospital MACE (OR 0.81, 95% CI 0.47–1.39; *p* = 0.444). However, propensity-score overlap was more limited, with approximately 10.3% of patients outside the common support, and the overall weighted effective sample size was 558. Residual covariate imbalance remained after weighting (maximum absolute SMD = 0.296), and the effective sample size within the CABG group was approximately 51. Accordingly, the CABG IPTW estimate was considered exploratory. No weight truncation was applied; maximum stabilized weights were 28.18 and 17.05 in the PCI and CABG comparisons, respectively. Covariate balance before and after IPTW for the no index revascularization–PCI and no index revascularization–CABG comparisons is presented in Supplementary Table S1.

With stabilized weights truncated at the 1st and 99th percentiles, the PCI versus no index revascularization estimate was OR 0.64 (95% CI 0.42–0.98; *p* = 0.039), whereas the CABG versus no index revascularization estimate was OR 0.94 (95% CI 0.55–1.62; *p* = 0.826). The PCI estimate therefore crossed the conventional threshold for statistical significance after weight truncation, while the CABG estimate remained non-significant. Given the observational design and sensitivity of the PCI estimate to weight handling, these findings were interpreted cautiously and did not alter the conclusions based on the primary multivariable analysis.

Missing-data sensitivity analysis: Peak troponin was missing in 866 of 1906 patients (45.4%) and was therefore excluded from the primary multivariable model. In a sensitivity analysis using the same multiple-imputation procedure but additionally including peak troponin, the principal treatment-strategy findings remained unchanged. Peak troponin was associated with in-hospital MACE (OR 1.019, 95% CI 1.007–1.030; *p* = 0.002), whereas neither PCI (OR 0.62, 95% CI 0.37–1.05; *p* = 0.074) nor CABG (OR 0.96, 95% CI 0.46–2.01; *p* = 0.915) was independently associated with MACE compared with no index revascularization. In the complete-case analysis, however, peak troponin was not significantly associated with MACE (OR 1.014, 95% CI 0.994–1.035; *p* = 0.167). These findings indicate that inclusion of peak troponin did not materially alter the associations between final management strategy and in-hospital MACE.

Among the 946 patients in the no index revascularization group, 181 (19.1%) met the study definition of MINOCA. After excluding these patients, 1725 patients remained (no index revascularization, *n* = 765; PCI, *n* = 709; CABG, *n* = 251). In-hospital MACE occurred in 87 (11.4%), 25 (3.5%), and 12 (4.8%) patients, respectively (*p* < 0.001). In a multivariable MICE sensitivity analysis excluding patients with MINOCA and using the same covariate set as the primary model, neither PCI (OR 0.67, 95% CI 0.39–1.14; *p* = 0.140) nor CABG (OR 0.89, 95% CI 0.43–1.84; *p* = 0.747) was independently associated with in-hospital MACE compared with no index revascularization. Exclusion of patients with MINOCA did not materially alter the treatment-group associations.

### 3.5. Surgical Characteristics of the CABG Group

Surgical details and in-hospital outcomes of the 251 CABG patients are presented in Table 4. Mean time to CABG was 10.3 ± 12.8 days, with a mean of 2.8 ± 1.0 grafts per patient. LIMA was utilized in 90.0% of patients, and RIMA use was documented in two patients (0.8%). Saphenous graft distribution included RCA in 58.6%, CX territory in 55.8%, diagonal in 34.3%, OM in 12.0%, and LAD in 9.2%. In-hospital mortality was 1.2%, and stroke occurred in 0.8% of patients.

## 4. Discussion

In this real-world cohort of 1906 NSTEMI patients undergoing coronary angiography, approximately half were managed with no index revascularization. The no index revascularization group had the highest baseline clinical risk and higher unadjusted in-hospital MACE and mortality. In the primary multivariable analysis, CKD, Killip class ≥2, lower LVEF, elevated CRP, and male sex were associated with in-hospital MACE, whereas neither PCI nor CABG was independently associated with MACE.

Importantly, because all patients in our cohort underwent coronary angiography, no index revascularization in the present study represents the absence of subsequent revascularization after angiographic assessment rather than an initially conservative strategy. Prior observational and pooled evidence indicates that a substantial proportion of patients with NSTEMI do not undergo coronary revascularization during the index hospitalization, reflecting advanced age, comorbidity burden, anatomical complexity, or patient-related factors [13,14]. The pronounced baseline differences between groups—no index revascularization patients being older, with higher CKD prevalence and lower LVEF—highlight substantial differences in baseline clinical risk and the potential for confounding by indication. Similarly, the higher prevalence of multivessel disease and LMCA involvement in the CABG group reflects marked differences in coronary anatomy across the final management pathways.

This distinction is important when interpreting our findings in the context of randomized trials comparing initial invasive and conservative strategies. In SENIOR-RITA, patients aged ≥75 years with NSTEMI were randomized to a routine invasive strategy or a conservative strategy, and routine invasive management did not significantly reduce the composite of cardiovascular death or nonfatal myocardial infarction [15]. In contrast, all patients in our cohort underwent coronary angiography before classification according to subsequent treatment. Therefore, our findings concern post-angiography treatment pathways and should not be interpreted as a comparison of invasive versus conservative management strategies.

The higher MACE rate in the no index revascularization group (9.8%) compared with PCI (3.5%) and CABG (4.8%) should be interpreted in the context of the substantially higher baseline risk of patients managed with no index revascularization and confounding by indication. The difference in composite MACE was primarily driven by all-cause mortality and cardiac arrest requiring CPR, whereas sustained VT/VF and stroke did not differ significantly among treatment groups. Given the heterogeneous severity and pathophysiology of the individual components, this study-specific composite should not be interpreted as a conventional cardiovascular MACE endpoint.

Importantly, 181 patients (19.1%) in the no index revascularization group met the study definition of MINOCA, highlighting the clinical heterogeneity of this management pathway. Exclusion of patients with MINOCA in the sensitivity analysis did not materially alter the associations between final management strategy and in-hospital MACE. Nevertheless, the remaining no index revascularization group likely included patients with heterogeneous coronary anatomy and clinical reasons for not undergoing revascularization. Therefore, this group should not be interpreted as representing a uniform medical-treatment strategy.

CKD emerged as the strongest independent factor associated with in-hospital MACE in the primary multivariable analysis (OR 3.70; 95% CI 2.36–5.81; *p* < 0.001). This finding is well-supported by the existing literature [4,16]. The adverse prognostic impact of renal dysfunction in ACS is multifactorial, encompassing uremic-toxin-mediated myocardial injury, anemia, volume overload, endothelial dysfunction, and underutilization of guideline-directed pharmacotherapy [17]. The GRACE risk score incorporates renal function as a major prognostic determinant [4]. The substantially higher CKD prevalence in the no index revascularization group (19.9% vs. 5.7% vs. 4.2%) further highlights marked differences in baseline renal risk across treatment groups. The higher incidence of dialysis-requiring AKI in the no index revascularization group may partly reflect its greater baseline renal vulnerability, including lower eGFR and a higher prevalence of CKD, rather than an effect of treatment strategy itself.

Lower LVEF was independently associated with in-hospital MACE (OR 0.97; 95% CI 0.95–0.99; *p* = 0.003), while Killip class ≥2 was associated with more than two-fold higher odds of MACE (OR 2.20; 95% CI 1.30–3.71; *p* = 0.003). These findings highlight the strong associations of ventricular dysfunction and clinical severity at presentation with adverse in-hospital outcomes. The prognostic value of Killip class reinforces the importance of hemodynamic status at presentation in NSTEMI [18]. Male sex was associated with higher odds of in-hospital MACE in the primary multivariable analysis. This finding should be interpreted cautiously, particularly because men were less represented in the no index revascularization group, which had the highest unadjusted event rate. Residual confounding or unmeasured differences in clinical characteristics may have contributed to this association, which should therefore be considered hypothesis-generating.

CRP was independently associated with in-hospital MACE in the primary multivariable model (OR 1.06; 95% CI 1.02–1.10; *p* = 0.003). Elevated CRP reflects the intensity of systemic inflammatory activity [4,19]. Peak troponin was excluded from the primary model because of substantial missingness (45.4%) and its potential temporal relationship with in-hospital management. In the MICE sensitivity analysis including peak troponin, it was associated with in-hospital MACE (OR 1.019; 95% CI 1.007–1.030; *p* = 0.002); however, this association was not significant in the complete-case analysis (OR 1.014; 95% CI 0.994–1.035; *p* = 0.167) [20]. Therefore, the association of peak troponin with in-hospital MACE should be interpreted cautiously. Importantly, inclusion of peak troponin did not materially alter the treatment-strategy associations. The reported CRP odds ratio corresponds to a 1 mg/L increase. Peak hs-cTnI values were reported in ng/mL; accordingly, the median peak hs-cTnI value of 0.15 ng/mL in the CABG group corresponds to 150 ng/L. This unit convention, together with the substantial missingness in peak hs-cTnI, should be considered when interpreting these values.

Neither PCI nor CABG was independently associated with in-hospital MACE in the primary multivariable model. The prespecified multivariable model with multiple imputation was considered the primary adjusted analysis because it was defined as the principal model for the full cohort, whereas IPTW analyses were performed as sensitivity analyses using an alternative adjustment framework. The PCI estimate became statistically significant after truncation of stabilized weights at the 1st and 99th percentiles. The PCI estimate was sensitive to the handling of extreme weights and should therefore be interpreted with caution. In the untruncated pairwise IPTW sensitivity analysis, PCI was not significantly associated with in-hospital MACE compared with no index revascularization (OR 0.71; 95% CI 0.48–1.06; *p* = 0.097). Although covariate balance improved substantially in this comparison, minor residual imbalance remained. The weighted CABG comparison was also non-significant (OR 0.81; 95% CI 0.47–1.39; *p* = 0.444); however, propensity-score overlap was more limited and residual covariate imbalance persisted. Therefore, the CABG IPTW analysis should be interpreted as exploratory. Overall, these findings indicate that a substantial proportion of the crude between-group differences may reflect baseline clinical risk and treatment selection rather than an independent effect of treatment strategy. Residual and unmeasured confounding remains possible because treatment allocation was not randomized.

The surgical revascularization profile of this cohort was characterized by LIMA use in 90.0% of CABG patients, whereas RIMA use was documented in only two patients; radial-artery use was not separately captured in the source dataset. These features should be considered when contextualizing the short-term surgical outcomes of this cohort.

The strengths of this study include the large real-world cohort, simultaneous evaluation of three treatment strategies within a single institution, multiple imputation to reduce loss of observations due to missing data, and propensity score-based sensitivity analysis. Several limitations should be acknowledged. The retrospective single-center design limits generalizability and precludes causal inference. Treatment allocation was non-random and influenced by clinical condition, coronary anatomy, comorbidity burden, and physician or Heart Team decision-making, resulting in confounding by indication. Although IPTW improved balance in measured characteristics in the no index revascularization–PCI comparison, residual confounding due to unmeasured factors cannot be excluded, and balance was less satisfactory for the no index revascularization–CABG comparison. The absence of SYNTAX score and long-term follow-up limits assessment of anatomical complexity and outcomes beyond hospitalization. Finally, the high proportion of missing peak troponin values (45.4%) introduces additional uncertainty. Peak troponin was therefore excluded from the primary multivariable model and evaluated only in sensitivity analyses. Moreover, because peak troponin represented the maximum value recorded during hospitalization and could have occurred after treatment allocation, its association should be interpreted cautiously and not as an independent pretreatment prognostic effect. Detailed information regarding troponin assay platforms and assay-specific reference limits across the entire study period could not be reliably reconstructed retrospectively. The no index revascularization group was clinically heterogeneous. Although patients with MINOCA could be identified and were addressed in a dedicated sensitivity analysis, detailed reasons for withholding revascularization and the exact proportion of the remaining patients with ≥50% coronary stenosis could not be reliably reconstructed from the retrospective dataset. Accordingly, the no index revascularization group should not be interpreted as representing a uniform medical-treatment strategy.

In addition, treatment groups were defined according to the eventual in-hospital treatment strategy. Because the precise timing of individual MACE components relative to angiography and definitive revascularization could not be reliably determined, immortal-time and reverse-causation bias cannot be excluded, particularly in patients awaiting CABG. Therefore, the study should be interpreted as a descriptive and adjusted associational comparison according to final in-hospital management strategy rather than as an estimate of the causal effects of PCI or CABG.

Furthermore, the marked anatomical differences between treatment groups, particularly in the CABG group, raise concerns regarding limited covariate overlap and the positivity assumption. Although IPTW substantially improved balance for the no index revascularization–PCI comparison, residual imbalance persisted in the no index revascularization–CABG comparison; therefore, the latter should be considered exploratory rather than causal. Importantly, IPTW does not eliminate this potential bias related to treatment-exposure timing.

Regarding missing-data handling, binary variables imputed using MICE were dichotomized at a threshold of 0.5, introducing a deterministic post-imputation step that may underestimate imputation-related uncertainty. Furthermore, missing covariate values in the propensity-score models were handled using median imputation rather than the MICE procedure used for the primary multivariable analysis. These methodological choices may have resulted in additional uncertainty in the adjusted estimates.

Detailed in-hospital and discharge pharmacotherapy data were not systematically available; therefore, differences in medical treatment between groups and their potential association with early outcomes could not be evaluated.

In addition, structured angiographic variables were incompletely available, particularly in the no index revascularization group, in which LMCA and multivessel-disease status were available for 536 and 535 of 946 patients, respectively. Although MINOCA status was recorded separately and was not derived solely from these variables, incomplete angiographic documentation and the need to impute missing angiographic covariates may have introduced additional uncertainty.

Finally, the six-year enrollment period (2020–2025) encompassed the COVID-19 pandemic and temporal changes in ACS management. Calendar-year effects were not specifically modeled, and residual confounding related to changes in clinical practice over time cannot be excluded. Frailty and formal surgical-risk measures were not systematically available, and potential diagnostic misclassification inherent to retrospective case ascertainment cannot be completely excluded.

## 5. Conclusions

Nearly half of NSTEMI patients undergoing coronary angiography in this real-world cohort were managed with no index revascularization, with substantially higher unadjusted in-hospital MACE and mortality compared to revascularized groups. CKD, Killip class ≥ 2, lower LVEF, elevated CRP, and male sex were independently associated with in-hospital MACE in the primary multivariable analysis. Neither PCI nor CABG was independently associated with MACE after adjustment. These findings underscore the prognostic associations of baseline clinical and laboratory characteristics in NSTEMI; however, their incremental prognostic value beyond established risk scores such as GRACE was not assessed in the present study.

## Figures and Tables

**Table 1:** Baseline demographic, clinical and angiographic characteristics.

Variable	No Index Revascularization (*n* = 946)	PCI (*n* = 709)	CABG (*n* = 251)	*p* Value
**Demographics**				
Age (years), mean ± SD	67.0 ± 13.9	59.7 ± 11.0	60.7 ± 10.0	<0.001
Male sex, n/N (%)	538/945 (56.9%)	557/709 (78.6%)	187/251 (74.5%)	<0.001
**Cardiovascular Risk Factors**				
Hypertension, n/N (%)	662/918 (72.1%)	368/686 (53.6%)	145/236 (61.4%)	<0.001
Diabetes mellitus, n/N (%)	408/940 (43.4%)	240/706 (34.0%)	97/251 (38.6%)	<0.001
Smoking, n/N (%)	256/913 (28.0%)	332/684 (48.5%)	93/236 (39.4%)	<0.001
**Comorbidities**				
COPD, n/N (%)	94/913 (10.3%)	32/683 (4.7%)	10/236 (4.2%)	<0.001
CKD, n/N (%)	182/916 (19.9%)	39/683 (5.7%)	10/236 (4.2%)	<0.001
Previous MI, n/N (%)	336/914 (36.8%)	181/689 (26.3%)	45/237 (19.0%)	<0.001
**Clinical Presentation**				
Killip class ≥ 2, n/N (%)	133/902 (14.7%)	21/669 (3.1%)	7/207 (3.4%)	<0.001
Heart rate (bpm), mean ± SD	90.8 ± 25.7 (*n* = 879)	80.8 ± 19.4 (*n* = 641)	84.4 ± 24.5 (*n* = 172)	<0.001
LVEF (%), mean ± SD	47.3 ± 12.1 (*n* = 852)	52.1 ± 9.4 (*n* = 623)	52.0 ± 9.6 (*n* = 228)	<0.001
**Laboratory Values**				
Serum creatinine (mg/dL), mean ± SD	1.37 ± 1.25 (*n* = 716)	1.02 ± 0.86 (*n* = 546)	0.98 ± 0.42 (*n* = 193)	<0.001
eGFR (mL/min/1.73 m^2^), mean ± SD	69.0 ± 30.4 (*n* = 460)	85.7 ± 20.6 (*n* = 421)	85.5 ± 20.1 (*n* = 175)	<0.001
CRP (mg/L), mean ± SD	3.3 ± 4.8 (*n* = 655)	1.8 ± 3.0 (*n* = 493)	2.6 ± 4.3 (*n* = 184)	<0.001
Hemoglobin (g/dL), mean ± SD	12.8 ± 2.2 (*n* = 717)	14.1 ± 1.7 (*n* = 548)	13.6 ± 1.9 (*n* = 197)	<0.001
Peak hs-cTnI (ng/mL), median [IQR]	1.00 [0.20–4.10] (*n* = 509)	1.17 [0.14–8.04] (*n* = 403)	0.15 [0.02–3.06] (*n* = 128)	0.003
**Angiographic Characteristics**				
Time to angiography (hours), mean ± SD	65.9 ± 72.2 (*n* = 142)	48.4 ± 46.9 (*n* = 175)	29.9 ± 37.5 (*n* = 56)	N/A
LMCA disease, n/N (%)	19/536 (3.5%)	12/697 (1.7%)	40/238 (16.8%)	<0.001
Multivessel disease, n/N (%)	177/535 (33.1%)	232/699 (33.2%)	214/238 (89.9%)	<0.001

Data are presented as mean ± SD, median [IQR], or n/N (%), as appropriate. For variables with missing observations, the number of available observations (*n*) is reported. For categorical variables, N represents the number of patients with available data. LVEF: left ventricular ejection fraction; eGFR: estimated glomerular filtration rate; CRP: C-reactive protein; LMCA: left main coronary artery; PCI: percutaneous coronary intervention; CABG: coronary artery bypass grafting; COPD: chronic obstructive pulmonary disease; MI: myocardial infarction; CKD: chronic kidney disease; N/A: not applicable.

**Table 2:** In-hospital outcomes according treatment strategy.

Outcome	No Index Revascularization (*n* = 946)	PCI (*n* = 709)	CABG (*n* = 251)	*p* Value
Primary endpoint (MACE), *n* (%)	93 (9.8%)	25 (3.5%)	12 (4.8%)	<0.001
All-cause mortality, *n* (%)	49 (5.2%)	3 (0.4%)	3 (1.2%)	<0.001
Cardiac arrest (requiring CPR), *n* (%)	54 (5.7%)	9 (1.3%)	2 (0.8%)	<0.001
Sustained VT/VF, *n* (%)	20 (2.1%)	11 (1.6%)	4 (1.6%)	0.742
Dialysis-requiring AKI, *n* (%)	30 (3.2%)	8 (1.1%)	4 (1.6%)	0.018
Stroke, *n* (%)	8 (0.8%)	3 (0.4%)	2 (0.8%)	0.620
In-hospital bleeding events, *n* (%)	5 (0.5%)	1 (0.1%)	Not evaluable	0.247
In-hospital atrial fibrillation, *n* (%)	41 (4.3%)	7 (1.0%)	Not evaluable	<0.001
Total hospital stay (days), mean ± SD	7.9 ± 8.2 (*n* = 240)	5.7 ± 3.4 (*n* = 176)	18.1 ± 10.1 (*n* = 57)	Not tested

MACE: composite of all-cause mortality, cardiac arrest requiring CPR, sustained VT/VF, dialysis-requiring AKI, and stroke during index hospitalization. VT/VF: ventricular tachycardia/fibrillation; AKI: acute kidney injury; CPR: cardiopulmonary resuscitation; PCI: percutaneous coronary intervention; CABG: coronary artery bypass grafting. Fisher’s exact test was used for sparse contingency tables, including sustained VT/VF, stroke, and in-hospital bleeding events; Pearson’s chi-square test was used for the remaining categorical comparisons, as appropriate. For in-hospital bleeding and atrial fibrillation, *p* values compare only no index revascularization with PCI (two-sided Fisher’s exact test for bleeding and Pearson’s chi-square test for atrial fibrillation). CABG data for these outcomes were not evaluable because of possible underascertainment in the retrospective dataset. Hospital stay is reported descriptively; no inferential comparison was performed.

**Table 3:** Multivariable logistic regression analysis for in-hospital MACE (MICE method).

Variable	OR	95% CI	*p* Value
Age (per 1-year increase)	1.01	1.00–1.03	0.161
**Male sex**	**1.63**	**1.04–2.58**	**0.035**
HT	1.10	0.68–1.77	0.700
DM	0.79	0.52–1.22	0.287
**CKD**	**3.70**	**2.36–5.81**	**<0.001**
**Left ventricular EF (%)**	**0.97**	**0.95–0.99**	**0.003**
**Killip class ≥ 2**	**2.20**	**1.30–3.71**	**0.003**
**CRP (per 1 mg/L increase)**	**1.06**	**1.02–1.10**	**0.003**
LMCA disease	0.57	0.19–1.70	0.309
Multivessel disease	1.14	0.69–1.88	0.605
PCI strategy (vs. no index revascularization)	0.68	0.41–1.13	0.137
CABG strategy (vs. no index revascularization)	0.92	0.42–2.00	0.833

Statistically significant results (*p* < 0.05) are shown in bold. Analysis performed using MICE on all 1906 patients (130 MACE events, 6.8%); 20 imputation sets pooled using Rubin’s rules. Peak troponin was excluded from the primary multivariable model because of substantial missingness (45.4%) and was evaluated separately in sensitivity analyses. OR: odds ratio; CI: confidence interval; CKD: chronic kidney disease; CRP: C-reactive protein; EF: ejection fraction; HT: hypertension; DM: diabetes mellitus; LMCA: left main coronary artery; MACE: major adverse cardiovascular events; PCI: percutaneous coronary intervention; CABG: coronary artery bypass grafting. Reference category is the no index revascularization group.

**Table 4:** Surgical characteristics and in-hospital outcomes of the CABG group (*n* = 251).

Variable	*n*	% (*n* = 251)
**Surgical Characteristics**		
Time to CABG (days), mean ± SD	10.3 ± 12.8	—
Number of grafts, mean ± SD	2.8 ± 1.0	—
LIMA use	226	90.0%
RIMA use	2	0.8%
LAD saphenous graft	23	9.2%
Diagonal saphenous graft	86	34.3%
CX saphenous graft	140	55.8%
OM saphenous graft	30	12.0%
RCA saphenous graft	147	58.6%
IMA–saphenous combination	15	6.0%
**In-Hospital Outcomes**		
Stroke	2	0.8%
In-hospital mortality	3	1.2%

LIMA: left internal mammary artery; RIMA: right internal mammary artery; LAD: left anterior descending artery; CX: circumflex artery; OM: obtuse marginal; RCA: right coronary artery; IMA: internal mammary artery; CABG: coronary artery bypass grafting. Graft-specific variables are presented at the patient level and are not mutually exclusive; therefore, their counts should not be summed to derive the total number of grafts. RIMA use was documented in two patients; radial-artery use was not separately captured in the source dataset. The IMA–saphenous variable represents a grafting configuration rather than an additional graft category.

## Data Availability

The data presented in this study are available on request from the corresponding author.

## Supplementary Materials

The following supporting information can be downloaded at: https://www.mdpi.com/article/10.3390/jcm15207872/s1, Table S1. Covariate balance before and after inverse probability of treatment weighting.

## Abbreviations

The following abbreviations are used in this manuscript:

NSTEMI	Non-ST-segment elevation myocardial infarction
MACE	Major adverse cardiovascular events
PCI	Percutaneous coronary intervention
CABG	Coronary artery bypass grafting
CKD	Chronic kidney disease
LVEF	Left ventricular ejection fraction
CRP	C-reactive protein
eGFR	Estimated glomerular filtration rate
LMCA	Left main coronary artery
AKI	Acute kidney injury
CPR	Cardiopulmonary resuscitation
VT/VF	Ventricular tachycardia/ventricular fibrillation
AF	Atrial fibrillation
COPD	Chronic obstructive pulmonary disease
MI	Myocardial infarction
MICE	Multiple imputation with chained equations
IPTW	Inverse probability of treatment weighting
SMD	Standardized mean difference

## References

[1] Timmis A., Kazakiewicz D., Townsend N., Huculeci R., Aboyans V., Vardas P. (2023). Global epidemiology of acute coronary syndromes. Nat. Rev. Cardiol..

[2] Cohen M., Visveswaran G. (2020). Defining and managing patients with non-ST-elevation myocardial infarction: Sorting through type 1 vs. other types. Clin. Cardiol..

[3] Bhatt D.L., Lopes R.D., Harrington R.A. (2022). Diagnosis and Treatment of Acute Coronary Syndromes: A Review. JAMA.

[4] Byrne R.A., Rossello X., Coughlan J.J., Barbato E., Berry C., Chieffo A., Claeys M.J., Dan G.A., Dweck M.R., Galbraith M. (2023). 2023 ESC Guidelines for the management of acute coronary syndromes. Eur. Heart J..

[5] Rao S.V., O’Donoghue M.L., Ruel M., Rab T., Tamis-Holland J.E., Alexander J.H., Baber U., Baker H., Cohen M.G., Cruz-Ruiz M. (2025). 2025 ACC/AHA/ACEP/NAEMSP/SCAI Guideline for the Management of Patients With Acute Coronary Syndromes: A Report of the American College of Cardiology/American Heart Association Joint Committee on Clinical Practice Guidelines. Circulation.

[6] Díez-Villanueva P., Jiménez-Méndez C., Cepas-Guillén P., Arenas-Loriente A., Fernández-Herrero I., García-Pardo H., Díez-Delhoyo F. (2024). Current Management of Non-ST-Segment Elevation Acute Coronary Syndrome. Biomedicines.

[7] Lee C.W., Ahn J.M., Cavalcante R., Sotomi Y., Onuma Y., Suwannasom P., Tenekecioglu E., Yun S.C., Park D.W., Kang S.J. (2016). Coronary Artery Bypass Surgery Versus Drug-Eluting Stent Implantation for Left Main or Multivessel Coronary Artery Disease: A Meta-Analysis of Individual Patient Data. JACC Cardiovasc. Interv..

[8] Huckaby L.V., Sultan I., Mulukutla S., Kliner D., Gleason T.G., Wang Y., Thoma F., Kilic A. (2020). Revascularization following non-ST elevation myocardial infarction in multivessel coronary disease. J. Card. Surg..

[9] Omerovic E., Råmunddal T., Petursson P., Angerås O., Rawshani A., Jha S., Skoglund K., Mohammad M.A., Persson J., Alfredsson J. (2025). Percutaneous vs. surgical revascularization of non-ST-segment elevation myocardial infarction with multivessel disease: The SWEDEHEART registry. Eur. Heart J..

[10] Matetic A., Mohamed M.O., Roberts D.J., Rana J.S., Alraies M.C., Patel B., Sauer A.J., Diaz-Arocutipa C., Sattar Y., Van Spall H.G.C. (2022). Real-world management and outcomes of 7 million patients with acute coronary syndrome according to clinical research trial enrolment status: A propensity matched analysis. Eur. Heart J. Qual. Care Clin. Outcomes.

[11] Mas-Llado C., Gonzalez-Del-Hoyo M., Siquier-Padilla J., Blaya-Peña L., Coughlan J.J., García de la Villa B., Peral V., Rossello X. (2023). Representativeness in randomised clinical trials supporting acute coronary syndrome guidelines. Eur. Heart J. Qual. Care Clin. Outcomes.

[12] Thygesen K., Alpert J.S., Jaffe A.S., Chaitman B.R., Bax J.J., Morrow D.A., White H.D. (2018). Fourth Universal Definition of Myocardial Infarction (2018). J. Am. Coll. Cardiol..

[13] van den Broek W.W.A., Gimbel M.E., Chan Pin Yin D., Azzahhafi J., Hermanides R.S., Runnett C., Storey R.F., Austin D., Oemrawsingh R., Cooke J. (2023). Conservative versus Invasive Strategy in Elderly Patients with Non-ST-Elevation Myocardial Infarction: Insights from the International Popular Age Registry. J. Clin. Med..

[14] Kotanidis C.P., Mills G.B., Bendz B., Berg E.S., Hildick-Smith D., Hirlekar G., Milasinovic D., Morici N., Myat A., Tegn N. (2024). Invasive vs. conservative management of older patients with non-ST-elevation acute coronary syndrome: Individual patient data meta-analysis. Eur. Heart J..

[15] Kunadian V., Mossop H., Shields C., Bardgett M., Watts P., Teare M.D., Pritchard J., Adams-Hall J., Runnett C., Ripley D.P. (2024). Invasive Treatment Strategy for Older Patients with Myocardial Infarction. N. Engl. J. Med..

[16] Weight N., Moledina S., Ullah M., Wijeysundera H.C., Davies S., Chew N.W.S., Lawson C., Khan S.U., Gale C.P., Rashid M. (2024). Impact of Chronic Kidney Disease on the Processes of Care and Long-Term Mortality of Non-ST-Segment-Elevation Myocardial Infarction: A Nationwide Cohort Study and Long-Term Follow-Up. J. Am. Heart Assoc..

[17] Curaj A., Vanholder R., Loscalzo J., Quach K., Wu Z., Jankowski V., Jankowski J. (2024). Cardiovascular Consequences of Uremic Metabolites: An Overview of the Involved Signaling Pathways. Circ. Res..

[18] Khot U.N., Jia G., Moliterno D.J., Lincoff A.M., Khot M.B., Harrington R.A., Topol E.J. (2003). Prognostic Importance of Physical Examination for Heart Failure in Non-ST-Elevation Acute Coronary Syndromes: The Enduring Value of Killip Classification. JAMA.

[19] Menekşe T.S., Kaçer İ., Hacımustafaoğlu M., Gül M., Ateş C. (2024). C-reactive protein to albumin ratio may predict in-hospital mortality in non-ST elevation myocardial infarction. Biomark. Med..

[20] Loutati R., Bruoha S., Taha L., Karmi M., Perel N., Maller T., Sabouret P., Galli M., Zoccai G.B., De Rosa S. (2024). Association between peak troponin level and prognosis among patients admitted to intensive cardiovascular care unit. Int. J. Cardiol..

