# Supplementary material for: In-Hospital Outcomes According to Treatment Strategy in NSTEMI Patients Undergoing Coronary Angiography: No Index Revascularization, PCI, or CABG?
Source: J Clin Med. 2026 Oct 13;15(20):7872. doi: 10.3390/jcm15207872 (PMC13706295; doi:10.3390/jcm15207872)
Supplement: Supplementary file 1 [file jcm-15-07872-s001.zip › jcm-4500659-supplementary.pdf]

Supplementary Table S1. Covariate balance before and after inverse probability of treatment weighting

Covariate	No index revascularization–PCI		No index revascularization–CABG	
	Before IPTW	After IPTW	Before IPTW	After IPTW
Age	0.566	0.050	0.506	0.232
Male sex	0.475	0.080	0.376	0.100
Hypertension	0.377	0.049	0.199	0.100
Diabetes mellitus	0.192	0.045	0.091	0.040
COPD	0.211	0.033	0.236	0.088
CKD	0.427	0.100	0.490	0.075
Previous MI	0.218	0.077	0.406	0.135
Current smoking	0.418	0.019	0.215	0.006
Killip class ≥2	0.406	0.115	0.414	0.289
Heart rate	0.423	0.075	0.264	0.069
LVEF	0.421	0.054	0.416	0.132
Serum creatinine	0.278	0.058	0.358	0.168
eGFR	0.420	0.004	0.458	0.089
CRP	0.313	0.030	0.109	0.161
Hemoglobin	0.568	0.056	0.321	0.142
LMCA disease	0.023	0.006	0.502	0.056
Multivessel disease	0.314	0.002	0.696	0.296

Values are absolute standardized mean differences (SMDs). An absolute SMD <0.10 was considered indicative of adequate covariate balance. Pairwise propensity-score models were constructed separately for PCI versus no index revascularization and CABG versus no index revascularization. Stabilized average-treatment-effect weights were used without truncation. The maximum post-weighting absolute SMD was 0.115 for the PCI comparison and 0.296 for the CABG comparison. Overall weighted effective sample sizes were 908 and 558, respectively; the weighted CABG-group effective sample size was approximately 51. Maximum stabilized weights were 28.18 and 17.05, respectively.
